# Two Cases of Bacteriemia Caused by Nontoxigenic, Non-O1, Non-O139 Vibrio cholerae Isolates in Ho Chi Minh City, Vietnam

**DOI:** 10.1128/JCM.01915-14

**Published:** 2014-10

**Authors:** Nguyen Phu Huong Lan, Tran Vu Thieu Nga, Nguyen Thi Thu Yen, Le Thi Dung, Ha Thanh Tuyen, James I. Campbell, Jamie Whitehorn, Guy Thwaites, Nguyen Van Vinh Chau, Stephen Baker

**Affiliations:** a The Hospital for Tropical Diseases, Ho Chi Minh City, Vietnam; b The Hospital for Tropical Diseases, Wellcome Trust Major Overseas Programme, Oxford University Clinical Research Unit, Ho Chi Minh City, Vietnam; c Centre for Tropical Medicine, Nuffield Department of Clinical Medicine, Oxford University, Oxford, United Kingdom; d The London School of Hygiene and Tropical Medicine, London, United Kingdom

## Abstract

The toxigenic bacterium Vibrio cholerae belonging to the O1 and O139 serogroups is commonly associated with epidemic diarrhea in tropical settings; other diseases caused by this environmental pathogen are seldom identified. Here we report two unassociated cases of nonfatal, nontoxigenic V. cholerae non-O1, non-O139 bacteremia in patients with comorbidities in Ho Chi Minh City, Vietnam, that occurred within a 4-week period.

## CASE REPORT

A 63-year-old female patient was admitted to the Hospital for Tropical Diseases (HTD) in Ho Chi Minh City (HCMC), Vietnam, in June 2013. She recalled a 4-week history of fatigue with loss of appetite, and she had developed jaundice with fever in the week prior to admission. The patient had a history of hypertension but no history of liver disease. On admission, she was fully conscious, afebrile, and hemodynamically stable; her respiratory rate was 20 breaths per minute. She had severe icterus, palmar erythema, and peripheral edema, and her liver and spleen were not palpable. She had detectable ascites (grade 2) but without abdominal tenderness or portal vein thrombosis. The patient's initial laboratory results are shown in Table 1, and her viral hepatitis serology results were as follows: anti-hepatitis A virus (anti-HAV) (IgM), negative; anti-hepatitis B virus (anti-HBV), negative; anti-HBc (IgM), negative; anti-hepatitis C virus (anti-HCV), negative; and HBsAg, positive. She had a quantitative HBV PCR blood result of 1 ×10^6^ copies/ml (Abbott Real-time HBV kit). The initially prescribed treatments were entecavir (0.5 mg once a day), rabeprazol (20 mg twice a day), diphenyl dimethyl bicarboxylate (DDB) (25 mg thrice a day), furosemide (25 mg once a day), and losartan (50 mg once a day). On the third day of admission, the patient's temperature peaked at 40°C with associated chills, nausea, and dizziness but without diarrhea or abdominal pain. Her procalcitonin level was elevated at 0.72 ng/ml. A bacterial infection was suspected, and 2 g/day of intravenous ceftriaxone was added to the medications.

**TABLE 1:** Initial laboratory test results of two patients with Vibrio cholerae non-O1, non-O139 bacteremia

Laboratory test^*a*^	Value(s)		
	Normal range	Patient 1	Patient 2
White cell count (×10^3^ cells/μl)	6–10	7.37	1.75
Polymorphonuclear cell (%)	49.6–71.3	58.4	83.2
Lymphocyte (%)	27.8–42.2	34.4	14.2
Monocyte (%)	0.2–4.3	18.2	0.8
Red cell count (×10^6^ cells/μl)	4.7–5.4	4.0	3.9
Platelet count (×10^3^ cells/μl)	201–324	160	37
AST (IU/liter)	<37	2,983	121
ALT (IU/liter)	<40	3,004	56
Total bilirubin (μmol/liter)	0–17	334.5	2.1
Direct bilirubin (μmol/liter)	0–4.3	272	3.2
Albumin (g/liter)	35–50	27.9	29.6
Prothrombin time (s)	11–13.5	23.9	18.1
TQ (%)	70–130	35	55
International normalized ratio	0.85–1.15	2.22	1.48

a ALT, alanine aminotransferase; AST, aspartate transaminase; TQ, temps de quick (prothrombin ratio).

An aerobic BacT/Alert bottle was used for a blood culture and incubated in a Bactec 9240 system (Becton Dickinson). The contents of the incubated bottle became positive after 12 h. A Gram stain on the positive BacT/Alert bottle revealed small curved Gram-negative bacilli, which were subcultured onto blood and MacConkey agar plates. The colonies displayed hemolysis on the blood agar plates and were oxidase positive. API20E and VITEK2 identification (bioMérieux, France) confirmed the organism to be Vibrio cholerae. Results of slide agglutination tests performed with polyvalent O1 and O139 antisera were negative. Antimicrobial susceptibility testing was performed by disc diffusion, and the results were interpreted according to the Clinical and Laboratory Standards Institute (CLSI) guidelines (1). The isolate was resistant to trimethoprim-sulfamethoxazole and tetracycline and susceptible to chloramphenicol, ofloxacin, ciprofloxacin, azithromycin, and ceftriaxone. The patient became afebrile after 2 days of ceftriaxone treatment, and yet the antimicrobial therapy was continued, with the other treatments, for an additional 8 days. The patient was ultimately discharged after 24 days of hospitalization.

In July 2013, a 73-year-old man was admitted to HTD with fever and confusion. He had been diagnosed with severe cirrhosis due to hepatitis C infection in a private health care facility 4 years previously and was actively receiving an unspecified treatment regimen. He had been unwell for 4 days with fever and constipation. Initial examination on the day of admission to hospital revealed the man to be thin, pale, and icteric, with peripheral edema and spider angiomata. His pulse was 97 bpm, his blood pressure was 140/80 mm Hg, and his respiratory rate was 24 breaths per minute. He was febrile, with a temperature of 39°C. His mental state was confused, and he was somnolent and had amnesia. A chest X-ray suggested that the patient had pneumonia, and an abdominal examination showed marked ascites with tenderness. An abdominal ultrasound revealed large amount of ascitic fluid and splenomegaly. A bacterial infection was suspected; therefore, the patient was prescribed 2 g of intravenous ceftriaxone/day, along with metronidazole (250 mg/day), furosemide (25 mg/day), and lactulose.

A yellow sample of ascitic fluid was drawn which was Rivalta test negative and negative for bacteria by Gram staining on microscopy. The fluid had 629 leukocytes/mm^3^ (86% neutrophils and 14% lymphocytes), 1,000 erythrocytes/mm^3^, 9 g/liter of protein, and 5.8 g/liter of albumin. The ascitic fluid was cultured on blood agar and MacConkey agar, and an aerobic BacT/Alert tube was taken for blood culture. Gram-negative curved bacilli were found both in the blood and the ascitic fluid isolated after overnight incubation. The organism in both samples was identified as V. cholerae, and neither sample agglutinated with O1 and O139 antisera. Antimicrobial susceptibility testing demonstrated that the organism was susceptible to all tested antimicrobials (ampicillin, chloramphenicol, ciprofloxacin, ceftriaxone, ofloxacin, trimethoprim-sulfamethoxazole, and tetracycline). The patient became afebrile after 2 days of ceftriaxone but was transferred to another hospital for surgical intervention after being diagnosed with bleeding of the upper gastrointestinal tract.

For confirmation of the microbiological identification, DNA preparations from the isolates from both patients were subjected to established PCR amplification methods targeting the rRNA intergenic spacer region of V. cholerae (2), the cholera toxin (CT) *ctxA* gene, the O1 O-antigen, and the O139 O-antigen (3). A toxigenic V. cholerae strain previously cultured from stool of a diarrheal patient was used as a control for the assays. All three of the isolates (two from blood and one from ascitic fluid) were PCR amplification positive for the rRNA intergenic spacer, confirming their microbiological identification as V. cholerae. All of the isolates were PCR amplification negative for the O139 antigen, the O1 antigen, and the *ctxA* toxin gene. We hence concluded the isolates to be nontoxigenic, non-O1, non-O139 V. cholerae.

Here we report two nonfatal cases of bacteremia caused by nontoxigenic V. cholerae, an atypical manifestation of this sometime pathogenic aquatic bacterial species. There are over 200 different reported serogroups of V. cholerae (4), but not all are capable of causing cholera. In fact, only CT-producing V. cholerae strains belonging to the serogroups O1 and O139 are associated with epidemic cholera (5, 6). However, other serogroups usually referred as non-O1 and non-O139 strains are occasionally reported to cause systematic infections. Patients with chronic syndromes, such as cirrhosis, hematologic abnormalities, renal dialysis, organ transplants, and immunosuppression, appear to be at increased risk of V. cholerae non-O1, non-O139 infections (7–11). Previous reports of retrospective studies originating from Taiwan (7) and Thailand (12) have described patients with cirrhosis and nontoxigenic Vibrio cholerae non-O1, non-O139 septicemia. Spontaneous peritonitis has also been observed in patients with Vibrio cholerae non-O1, non-O139 septicemia (13). The more typical manifestations of this infection are ascites, fever, jaundice, diarrhea, skin lesions, and gastrointestinal bleeding (13, 14), and we observed the majority of these symptoms in the patients described in this report. However, we observed no skin lesions or cellulitis. It has been reported that non-O1, non-O139 V. cholerae infections are associated with consumption of, or contact with, raw seafood, which is a risk factor similar to that for the related Vibrio species V. parahaemolyticus (15). However, is also noteworthy that neither of the patients whose cases are described here reported contact with, or consumption of, seafood.

The management of Vibrio cholerae non-O1, non-O139 infections differs substantially from management of epidemic diarrhea. The role of antimicrobials in severe cholera is not as critical as that of fluid and electrolyte replacement. In contrast, antimicrobials are essential for the management of extragastrointestinal Vibrio infections; however, there are currently no standard guidelines for treating this disseminated infection. Therefore, assessing the antimicrobial susceptibility pattern of the infecting Vibrio sp. is paramount for tailoring treatment. Currently, Vibrio cholerae non-O1, non-O139 isolates from many locations are still reported to be susceptible to beta lactams, fluoroquinolones, trimethoprim-sulfamethoxazole, tetracycline, and chloramphenicol (1, 3, 8, 9, 10). It has been suggested that third-generation cephalosporins or fluoroquinolones are the most suitable agents for treating V. cholerae septicemia (1). Indeed, ciprofloxacin seems to be associated with a favorable outcome, and we can report here that both patients described in this report recovered from the bloodstream infection quickly with a good clinical response after ceftriaxone treatment.

This is the first report of nontoxigenic V. cholerae bacteremia in Vietnam, a country in which the prevalence of hepatitis B virus infection is high. This high prevalence of hepatitis in Vietnam predicts increasing numbers of nontoxigenic V. cholerae infections in the future. We suggest that clinicians should consider these organisms alongside the more common agents of bacteremia in diagnoses of cirrhosis patients in tropical settings.
